# Punicalagin Protects against Diabetic Liver Injury by Upregulating Mitophagy and Antioxidant Enzyme Activities

**DOI:** 10.3390/nu14142782

**Published:** 2022-07-06

**Authors:** Yahui Zhang, Xiuying Tan, Yuan Cao, Xin An, Jihua Chen, Lina Yang

**Affiliations:** Xiangya School of Public Health, Central South University, Changsha 410128, China; yahuizhang@csu.edu.cn (Y.Z.); xiuyingtan@csu.edu.cn (X.T.); caoyuan2017@csu.edu.cn (Y.C.); 2501130307@csu.edu.cn (X.A.); chenjh@csu.edu.cn (J.C.)

**Keywords:** punicalagin, diabetic liver injury, oxidative stress, mitophagy, MnSOD, CAT

## Abstract

Diabetic liver injury has received increasing attention as a serious complication of type 2 diabetes. Punicalagin (PU), a major component of pomegranate polyphenols, has various biological activities such as antioxidant, anti-inflammatory, and lipid metabolism regulation. In this study, we observed the protective effect of punicalagin on a high-fat diet (HFD) and streptozotocin (STZ)-induced diabetic liver injury in mice and revealed the underlying mechanism. The results showed that fasting blood glucose (FBG), fasting serum insulin (FINS), and homeostasis model assessment for insulin resistance (HOMA-IR) in diabetic liver injury mice were significantly decreased after punicalagin intervention. Simultaneously, the levels of alanine aminotransferase (ALT), aspartate aminotransferase (AST), total cholesterol (TC), triglyceride (TG), low-density lipoprotein cholesterol (LDL-C), free fatty acids (FFA), malondialdehyde (MDA), and total superoxide dismutase (T-SOD) in the serum and liver were significantly decreased, with reductions in fat lesions and inflammatory cells. Mitophagy is a selective autophagy that maintains a balance between the quality and quantity of intracellular mitochondria. Studies have shown that mitophagy is closely related to the occurrence and development of diabetic liver injury. In our study, the mitochondrial membrane potential (MMP) was significantly increased in mice with diabetic liver injury after punicalagin intervention; the protein expression of Pink1, Parkin, Bnip3, LC3b, P62, manganese superoxide dismutase (MnSOD), and catalase (CAT) was significantly increased in the liver; and the activities of MnSOD and CAT in the serum and liver were significantly increased, which is consistent with the results of in vitro experiments. In summary, our study provided evidence that punicalagin could reduce the level of oxidative stress in the liver by upregulating mitophagy and the activities of antioxidant enzymes, thus having a certain protective effect against diabetic liver injury.

## 1. Introduction

Type 2 diabetes mellitus (T2DM) is a chronic and complex metabolic disease characterized by hyperglycemia. The latest version of the International Diabetes Federation (IDF) map data indicated that, in 2021, about 537 million adults worldwide would suffered from diabetes, with an age-adjusted prevalence rate of 9.8% [1]. The liver is an important tissue organ that regulates glucose and lipid metabolism in the body. The increased production of advanced glycation end products (AGE) caused by hyperglycemia and the accumulation of free fatty acids (FFA) in the liver caused by dyslipidemia can all lead to diabetic liver injuries, such as fatty liver, cirrhosis, and hepatocellular carcinoma [2,3]. In addition, diabetic liver injury can further aggravate the disorder of glucose and lipid metabolism in patients with type 2 diabetes, forming a vicious circle [2,3]. As a serious complication of type 2 diabetes, diabetic liver injury has received increasing attention.

To date, the pathogenesis of diabetic liver injury has not been fully understood. Recent studies have found that oxidative stress plays a key role in this process [4]. Regarding how to alleviate the level of oxidative stress, on the one hand, studies have shown that the decline in antioxidant capacity is closely related to the imbalance of intracellular oxidative stress. Antioxidative enzymes such as manganese superoxide dismutase (MnSOD) and catalase (CAT), as the first line of defense against oxidation in cells, can remove excess ROS, thereby maintaining the intracellular redox balance. On the other hand, mitochondrial damage is also closely related to intracellular oxidative stress. Excessive ROS can lead to mitochondrial swelling and the opening of the mitochondrial permeability transition pore (mPTP) [5], resulting in a large number of damaged mitochondria, which in turn can further promote the generation of ROS and aggravate the occurrence of oxidative stress [6].

In recent years, mitophagy has been gradually discovered as a selective autophagy, which is accompanied by a decrease in MMP and the opening of the mPTP. During mammalian mitophagy, in addition to the classic PINK1/Parkin-related mitophagy receptors, mitophagy receptors such as AMBRA1, FUNDC1, and Nix/BNIP3L were also found [7,8,9]. Recent studies have shown that mitophagy plays an important role in the occurrence and development of chronic liver diseases. Shao et al. showed that the levels of mitophagy-related proteins Parkin and BNIP3L were significantly reduced in non-alcoholic fatty liver model mice, while exenatide intervention increased the expression of Parkin and BNIP3L and the number of autophagosomes, thereby attenuating oxidative stress damage and protecting against non-alcoholic fatty liver and diabetic liver damage [10]. Zhou et al. found that macrophage stimulation 1 (Mst1) inhibited Parkin-related mitophagy activity, thereby downregulating hepatic antioxidant factors such as superoxide dismutase (SOD) and glutathione peroxidase (GSH-PX), and increasing oxidative products such as GSSG and MDA [11]. In conclusion, an increasing intracellular antioxidant enzyme level and regulating intracellular mitophagy are particularly important for improving oxidative stress and may be an important target for the protection of diabetic liver injury.

Polyphenols are natural antioxidants found in a variety of human dietary components, such as fruits, vegetables, and tea, and have a protective effect against a variety of chronic diseases, including diabetes, cardiovascular diseases, and neurodegenerative diseases [12]. Punicalagin (PU) is a hydrolyzable tannin extracted from pomegranate peel polyphenols, which often adopts antioxidant, anti-inflammatory, and lipid metabolism regulation functions through hydrolysis to ellagic acid and urolithins in vivo [13,14,15], thus having a certain protective effect against diseases such as diabetic liver injury, cardiovascular disease, and prostate cancer [16,17]. Based on the powerful antioxidant properties of punicalagin, it is suggested that punicalagin may alleviate the occurrence and development of diabetic liver injury by improving oxidative stress. Meanwhile, studies have shown that punicalagin is involved in the regulation of autophagy [12,18,19], but there is no relevant research on the role of mitophagy, a selective autophagy, in diabetic liver injury.

In conclusion, this study selected punicalagin for intervention and verified the protective effect of punicalagin on diabetic liver injury through in vitro and in vivo experiments. The possible mechanisms of punicalagin’s protective effect were discussed from the perspectives of mitophagy and antioxidant enzyme system, which provided new ideas for the development and utilization of punicalagin and the prevention and treatment of diabetic liver injury.

## 2. Materials and Methods

### 2.1. Reagents and Antibodies

Punicalagin (CAS: 65995.63.3, 98% purity) was purchased from Herbpurify Co., Ltd. (Chengdu, China). Total cholesterol (TC), triglyceride (TG), alanine aminotransferase (ALT), aspartate aminotransferase (AST), low-density lipoprotein cholesterol (LDL-C), free fatty acids (FFA), malondialdehyde (MDA), total superoxide dismutase (T-SOD), CAT and MnSOD assay kits were purchased from the Jiancheng Bioengineering Institute (Nanjing, China). SANNUO blood glucose meter and test strips were purchased from Sannuo Biosensing Co., Ltd. (Changsha, China). Fasting serum insulin (FINS) ELISA kits were purchased from CUSABIO (Wuhan, China). An MMP assay kit with JC-1 and a tissue mitochondria isolation kit were purchased from Beyotime Biotechnology (Shanghai, China). Streptozotocin (STZ) was purchased from Solarbio Science & Technology Co., Ltd. (Beijing, China). Antibodies targeting the following proteins were used in this study: Parkin (PB0342, BOSTER), Pink1 (A11435, Abclonal), Bnip3 (A5683, Abclonal), LC3b (12741, Cell Signaling Technology), P62 (18420-1-A, Proteintech), MnSOD (PB0454, BOSTER), CAT (21260-1-AP, Proteintech), β-actin (60008-1-Ig, Proteintech). Horseradish peroxidase (HRP)-conjugated goat anti-mouse or anti-rabbit antibodies were purchased from ZSGB-BIO Biotechnology Co., Ltd. (Beijing, China). Membrane blots were developed using the BeyoECL Star system (Beyotime Biotechnology). All chemicals and solvents were of analytical grade. 

### 2.2. Animals and Treatment 

A total of 30 male C57BL/6 mice (20 ± 2 g, 6–8 weeks) were housed in an environment of room temperature 22 ± 2 °C, humidity 55-60%, and light conditions (12 h light/dark cycle). All the animals were free to drink and eat ad libitum. After one week of adaptive feeding, the mice were randomly divided into a control group, T2DM group, and T2DM + PU group. Mice in the T2DM group and T2DM + PU group were intraperitoneally injected with 100 mg/kg STZ dissolved in 0.1 mol/L citric acid buffer (pH = 4.4) after 8 weeks of a high-fat diet, while mice in the control group were intraperitoneally injected with equal volume citric acid buffer (pH = 4.4) after 8 weeks of normal feeding. Two days after injection of STZ, the fasting blood glucose (FBG) level of mice was measured, and ≥11.1 mmol/L was considered a successful type 2 diabetes mouse model. After successful modeling, mice in the T2DM + PU group were gavaged with PU once a day (20 mg/kg body weight/day) for 8 weeks, while mice in the control group and T2DM group were gavaged with the same volume of distilled water. Water intake, body weight, and blood glucose of mice were monitored during administration.

### 2.3. HOMA-IR

After the blood was collected from the tail vein, the fasting blood glucose level of the mice was measured with a blood glucose meter and test strips. Insulin content in mouse serum was detected by a mouse insulin ELISA detection kit. Homeostasis model assessment method - the calculation formula of insulin resistance index is: HOMA-IR = fasting insulin (μU/mL) × fasting glucose (mM)/22.5; HOMA-IS = 1/HOMA-IR.

### 2.4. Detection of Serum and Liver Biochemical Indicators

Mouse serum and liver samples were diluted to the appropriate concentrations, and biochemical kits were used to assay the levels of AST, ALT, TC, TG, LDL-C, FFA, MDA, T-SOD, MnSOD, and CAT.

### 2.5. H&E Staining

Liver samples fixed in 4% neutral paraformaldehyde solution were embedded in paraffin and sectioned. The sections were immersed in hematoxylin staining solution for 5–8 min and stained with acid water and ammonia for several seconds. After staining with eosin dye for 2–3 min, the sections were placed in different concentration gradients of ethanol and xylene to become transparent, and finally sealed with neutral gum. The sections were viewed under a microscope (MOTIC CHINA GROUP Co., Ltd., Xiamen, China) and the desired images were selected for acquisition and analysis.

### 2.6. Detection of Mitochondrial Membrane Potential

First, 1 mL of JC-1 working solution was added to the extracted mouse liver mitochondria. Excitation and emission light of JC-1 monomer (green light) were 490 nm and 530 nm, respectively, and for JC-1 complex (red light), were 525 nm and 590 nm, respectively. The membrane potential of liver mitochondria was expressed as the ratio of fluorescence intensity of red light to green light. In in vitro experiments, after the cells were treated, 1 mL of cell culture solution and 1 mL of JC-1 staining working solution were added, fully mixed, and then incubated in the cell incubator at 37 °C for 20 min. Finally, the cells were washed twice with JC-1 staining buffer (1×) and cell culture solution. Then, the cells were observed under a fluorescent microscope (EVOS M7000, Thermo Fisher Scientific, Waltham, MA, USA) and photographed.

### 2.7. Cell Culture and Grouping

Human hepatoma HepG2 cells were cultured in RPMI-1640 cell medium containing 1% penicillin-streptomycin and 10% fetal bovine serum in a cell culture incubator at 37 °C and 5% CO_2_. The morphology and growth density of the cells were observed under an inverted microscope, and the cells were subcultured every 2 to 4 days. In this study, HepG2 cells were divided into a control group, high-glucose (HG) group, and punicalagin group (low, medium, and high dose), and HepG2 cells in HG and punicalagin groups were cultured in a high-glucose environment for 48 h to simulate a type 2 diabetes model in vitro.

### 2.8. Western Blot Analysis

The separation gel concentration (10–15%) was determined by the molecular weight of the target protein. Furthermore, 10–50 mg of sample was added to the loading wells, and electrophoresis was stopped when the target band was completely separated. Gels containing target protein and internal reference were cut out and transferred. PVDF membranes were blocked in 5% skim milk powder for 1–2 h, incubated overnight at 4 °C by the corresponding primary antibody dilutions, and then incubated for 1–2 h by HRP-IgG secondary antibody. ECL luminescent solution was used for color development. A gel imager (Tanon Technology Co., Ltd., Shanghai, China) was used to capture the ECL. The strips were washed with TBS-T buffer before each step. The relative amount of target protein was expressed as the ratio of the gray value of the target band to the corresponding β-actin band.

### 2.9. Statistical Analysis

The IBM SPSS 26.0 software (IBM Corp., Armonk, NY, USA) was used for statistical analysis. GraphPad Prism 8.0 (GraphPad Software, Inc., San Diego, CA, USA) was used for graphing. All data are expressed as the mean ± SD. Data normality tests and homogeneity of variance tests were analyzed first. When the data were normally distributed and the variances were homogeneous, an unpaired Student’s *t*-test was used to compare the difference between two groups, and a one-way ANOVA was used for multiple group comparisons. Non-parametric tests were used for data that did not meet the above conditions. *p* < 0.05 was considered statistically significant.

## 3. Results

### 3.1. Effects of Punicalagin on T2DM

**Figure 1:**
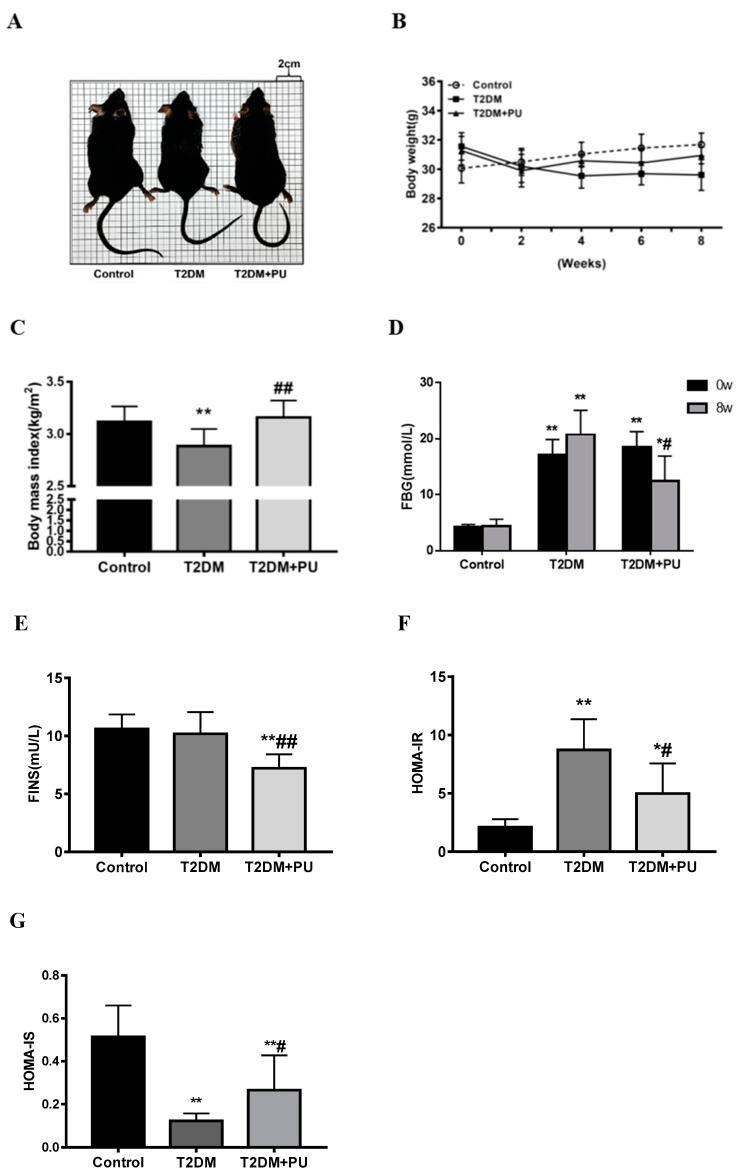
Effects of punicalagin on T2DM mice (*n* = 10). (**A**) Photos of mice in each group at the end of the experiment; (**B**) body weight; (**C**) BMI level; (**D**) FBG level; (**E**) FINS level; (**F**) HOMA-IR level; (**G**) HOMA-IS level. * *p* < 0.05 vs. Con group; ** *p* < 0.01 vs. Con group; # *p* < 0.05 vs. T2DM group; ## *p* < 0.01 vs. T2DM group.

As shown in Table 1, mice in the T2DM group developed typical symptoms of type 2 diabetes, such as mental fatigue and increased water intake, compared to the control group. Compared with the T2DM group, mice in the T2DM + PU group presented alleviation of type 2 diabetes symptoms, such as a significant decrease in water intake. Before STZ injection, the mean body weights of mice in the T2DM and T2DM + PU groups were similar, and both were higher than those of control mice. After STZ injection, the body weight of mice in the T2DM group gradually decreased, while that of the T2DM + PU group gradually decreased from 0 to 2 weeks and recovered from 2 to 8 weeks (see Figure 1A,B).

At the end of the experiment, BMI and HOMA-IS levels decreased significantly in the T2DM group compared with the control group, while FBG and HOMA-IR levels increased significantly (*p* < 0.01). Compared with the T2DM group, BMI and HOMA-IS levels were significantly increased in the T2DM + PU group, while FBG, FINS, and HOMA-IR levels were significantly decreased (*p* < 0.05, see Figure 1C–G). TC, TG, LDL-C, and FFA levels were increased in serum and liver homogenates of mice in the T2DM group compared with the control group (*p* < 0.05) and decreased in the T2DM + PU group compared with the T2DM group (*p*< 0.05, see Table 2). 

### 3.2. Punicalagin Protects Diabetic Liver Injury

**Figure 2:**
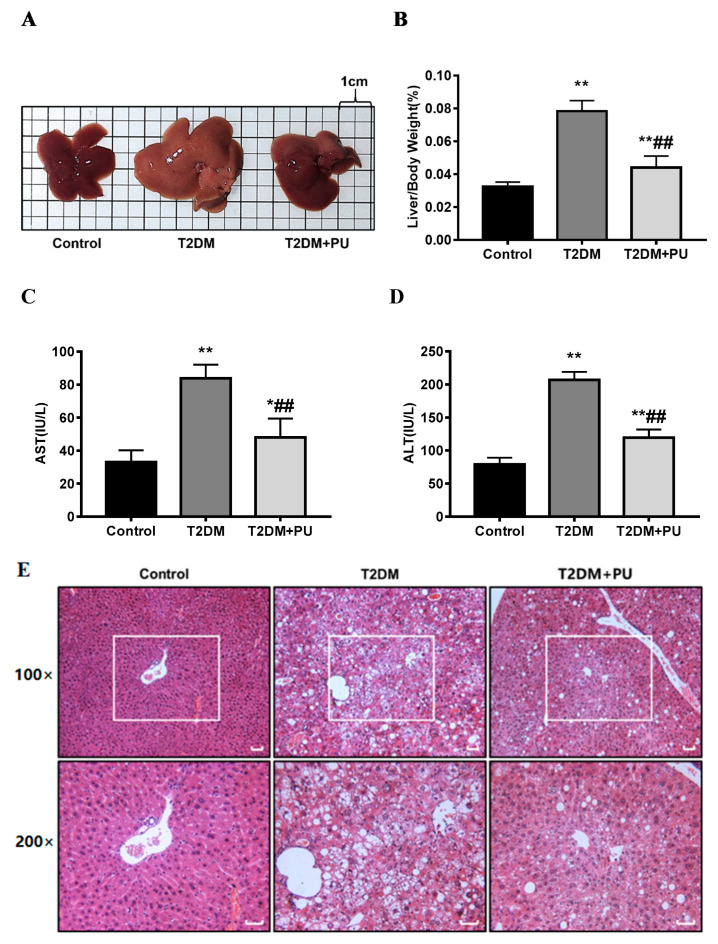
Effects of punicalagin on liver injury in T2DM mice (*n* = 10). (**A**) Liver morphology; (**B**) liver index; (**C**) serum AST level; (**D**) serum ALT level; (**E**) H&E-stained liver sections (scale bar = 200 μm). * *p* < 0.05 vs. Con group; ** *p* < 0.01 vs. Con group; ## *p* < 0.01 vs. T2DM group.

In terms of the liver morphology of mice, the livers of the control mice were reddish-brown, soft, and smooth, while the livers of the T2DM group were lighter in color and less soft. The liver color of the T2DM + PU group was similar to that of the control group (see Figure 2A). The liver index, and serum AST and ALT activity were significantly higher in the T2DM group compared with the control group (*p* < 0.01), while the liver index and ALT activity were significantly lower in the T2DM + PU group compared with the T2DM group (*p* < 0.01, see Figure 2B–D).

From the liver tissue structure of mice, the H&E staining results showed that the control mice had well-structured liver lobules, regularly shaped and well-arranged hepatocytes, and no hepatocyte steatosis or necrosis. Mice in the T2DM group had a disordered liver lobule structure, irregular hepatocyte morphology, obvious swelling, steatosis, and infiltration of multiple inflammatory cells. Compared with the T2DM group, the T2DM + PU group had a relatively clear and orderly cell structure, improved steatosis, and significantly fewer inflammatory cells (see Figure 2E).

### 3.3. Punicalagin Protects against Diabetic Liver Injury by Attenuating Oxidative Stress

**Figure 3:**
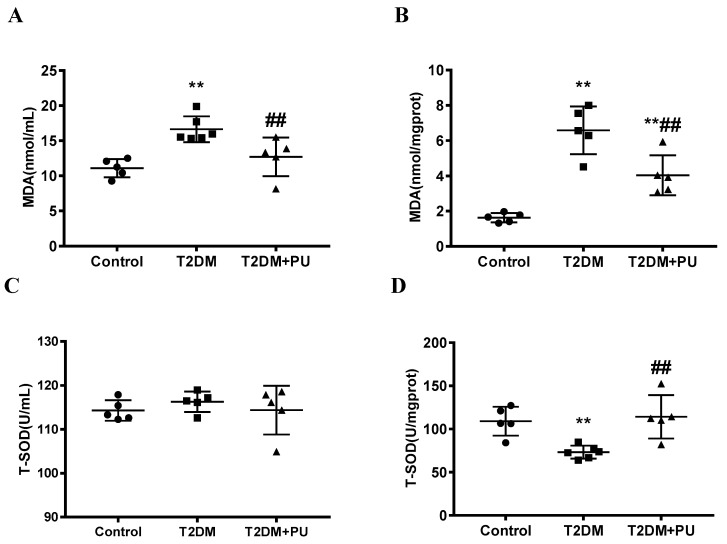
Effects of punicalagin on MDA and T-SOD levels in T2DM mice (*n* = 5–6). (**A**) Serum MDA; (**B**) MDA in liver homogenate; (**C**) serum T-SOD; (**D**) T-SOD in liver homogenate. ** *p* < 0.01 vs. Con group; ## *p* < 0.01 vs. T2DM group.

The oxidative stress level in the mouse liver was observed by detecting the levels of MDA and T-SOD in the serum and liver tissue in the research. Compared with the control group, MDA levels in the serum and liver were improved (*p* < 0.01) and T-SOD activity in the liver significantly declined (*p* < 0.01) in the T2DM group. Compared with the T2DM group, the MDA levels in the serum and liver of the T2DM + PU group were significantly decreased (*p* < 0.01), and the T-SOD activity in the liver was significantly increased (*p* < 0.01), while the T-SOD activity in serum was not changed (see Figure 3).

### 3.4. Punicalagin Regulates Mitophagy to Attenuate Oxidative Stress 

**Figure 4:**
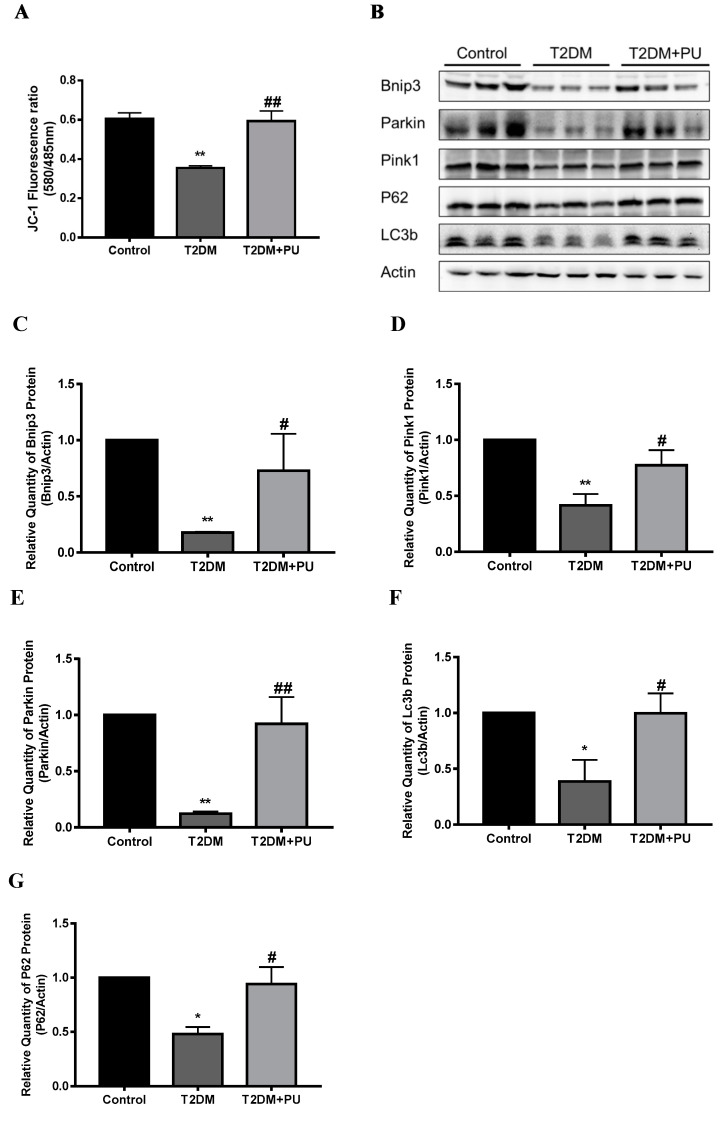
Effects of punicalagin on hepatic mitophagy in T2DM mice (*n* = 6). (**A**) MMP level; (**B**–**G**) the expression levels of Bnip3, Pink1, Parkin, LC3b, and P62 proteins. * *p* < 0.05 vs. Con group; ** *p* < 0.01 vs. Con group; # *p* < 0.05 vs. T2DM group; ## *p* < 0.01 vs. T2DM group.

Mitophagy was observed by detecting the MMP and the expression levels of mitophagy-related proteins. In the in vivo experiment, compared with the control group, the red/green fluorescence intensity was significantly decreased in the T2DM group (*p* < 0.01), and the MMP was 0.59 times that of the control group. Compared with the T2DM group, the red/green fluorescence intensity was significantly increased (*p* < 0.01) in the T2DM + PU group, and the decreasing trend of MMP was suppressed, which was 1.6 times higher than that of the T2DM group (see Figure 4A). The expressions of Bnip3, Pink1, Parkin, LC3b, and P62 proteins were reduced in the liver of mice in the T2DM group compared with the control group (*p* < 0.05). The expressions of Bnip3, Pink1, Parkin, LC3b, and P62 proteins were increased in the T2DM + PU group compared with the T2DM group (*p* < 0.05, see Figure 4B–G).

**Figure 5:**
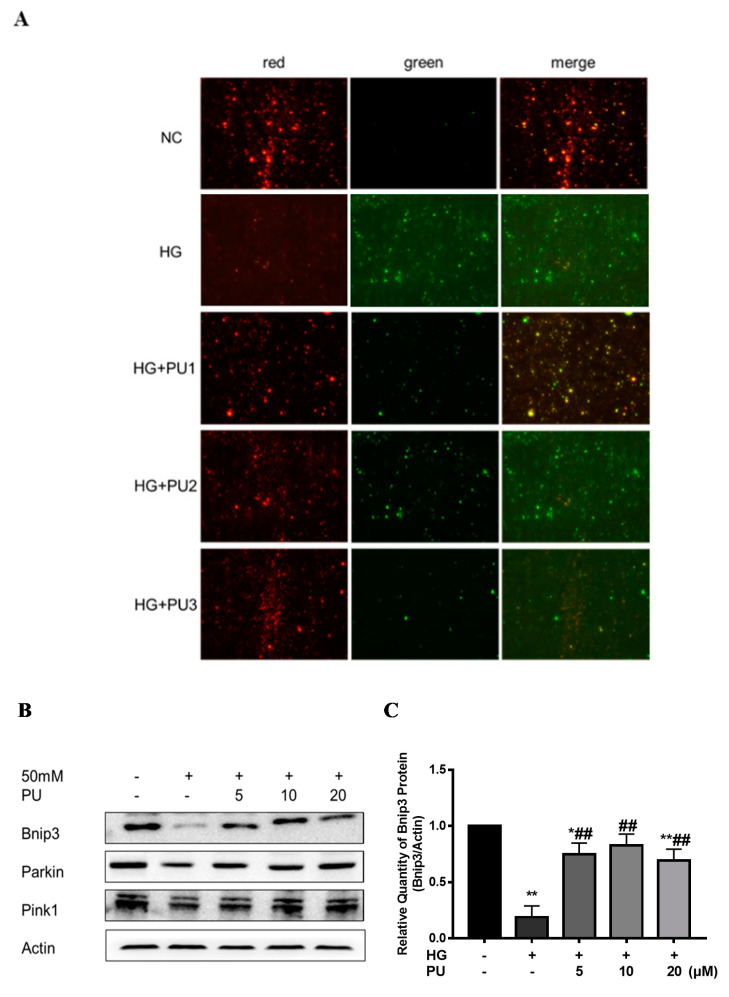

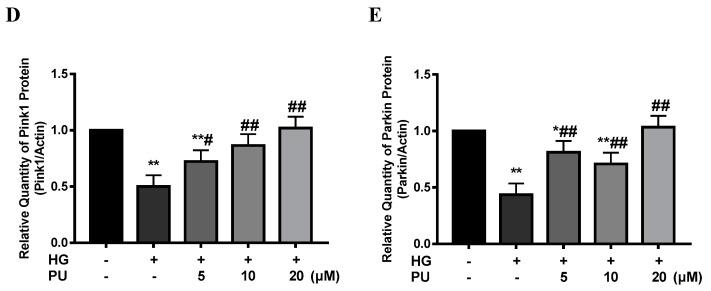
Effects of punicalagin on mitophagy in HepG2 cells in high−glucose environment (*n* = 3). (**A**) MMP level. (**B**–**E**) The expression levels of Bnip3, Pink1, and Parkin proteins. * *p* < 0.05 vs. control group; ** *p* < 0.01 vs. control group; # *p* < 0.05 vs. HG group; ## *p* < 0.01 vs. HG group.

In the in vitro experiments, the red/green fluorescence intensity was decreased in the HG group compared with the control group. However, after treating the cells with different concentrations (5, 10, 20 μM) of punicalagin for 48 h, the decreasing trend of MMP was suppressed and the red/green fluorescence intensity increased (see Figure 5A). As shown in Figure 5B–E, compared to the control group, the expression of Bnip3, Pink1, and Parkin proteins were reduced in the HG group (*p* < 0.05), while the intervention of punicalagin reversed the downward trend (*p* < 0.05). 

### 3.5. Punicalagin Regulates Antioxidant Enzyme System to Attenuate Oxidative Stress

**Figure 6:**
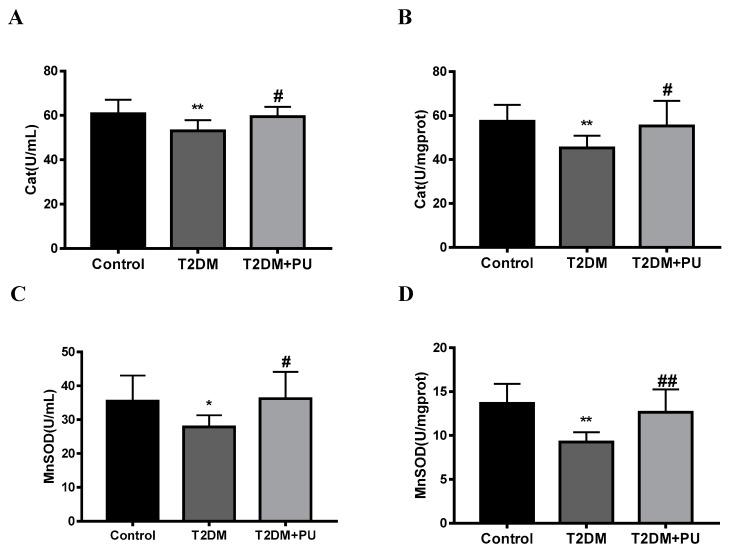

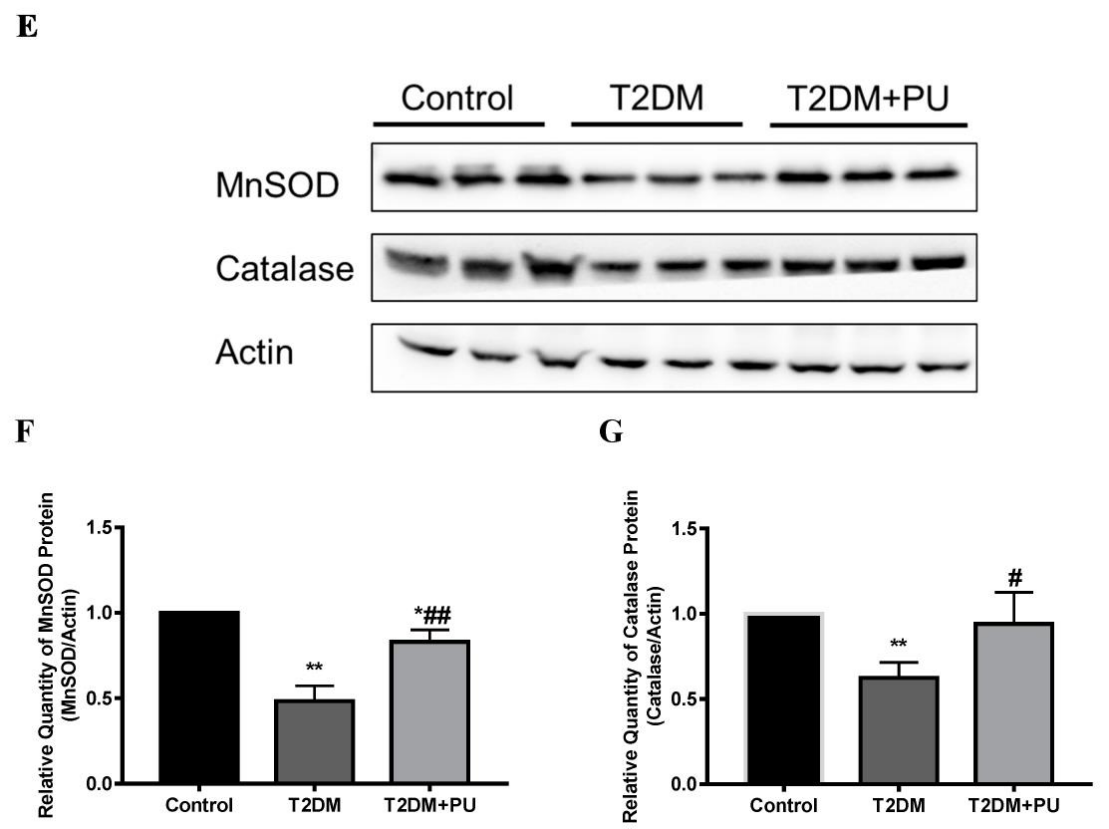
Effects of punicalagin on MnSOD and CAT levels in T2DM mice (*n* = 10). (**A**) Serum CAT; (**B**) liver homogenate CAT; (**C**) serum MnSOD; (**D**) liver homogenate MnSOD. (**E**–**G**) The expression levels of MnSOD and CAT proteins (*n* = 6). * *p* < 0.05 vs. Con group; ** *p* < 0.01 vs. Con group; # *p* < 0.05 vs. T2DM group; ## *p* < 0.01 vs. T2DM group.

In the in vivo experiment, compared with the control group, the activities of MnSOD and CAT in the serum and liver were decreased in the T2DM group (*p* < 0.05), while the activities of MnSOD and CAT in the serum and liver were increased in the T2DM + PU group compared with the T2DM group (*p* < 0.05, see Figure 6A–D). As shown in Figure 6E–G, compared with the control group, the expression levels of MnSOD and CAT proteins were significantly reduced in the liver of the T2DM group (*p* < 0.01), whereas the expression levels of MnSOD and CAT proteins were increased after punicalagin intervention (*p* < 0.05).

**Figure 7:**
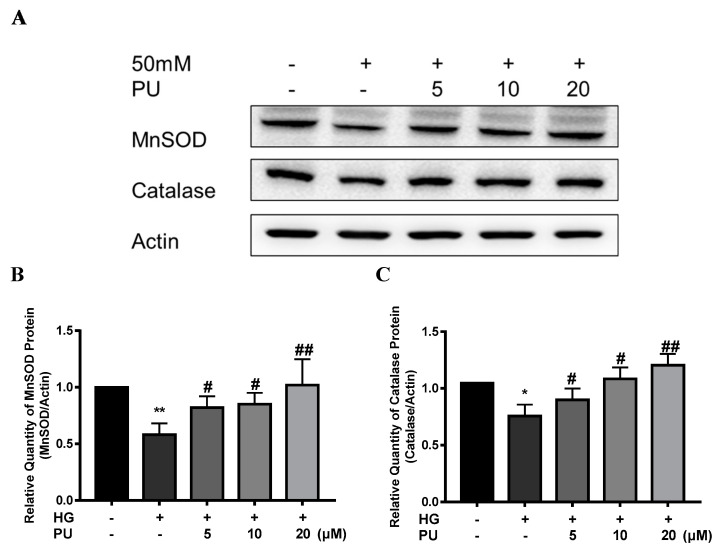
Effects of punicalagin on MnSOD and CAT protein expression in HepG2 cells in high−glucose environment (*n* = 3). (**A**–**C**) The expression levels of MnSOD and CAT proteins. * *p* < 0.05 vs. control group; ** *p* < 0.01 vs. control group; # *p* < 0.05 vs. HG group; ## *p* < 0.01 vs. HG group.

In addition, compared with the control group, the expressions of MnSOD and CAT proteins were decreased in the HG group in the in vitro experiment (*p* < 0.05), while the intervention of punicalagin reversed the downward trend (*p* < 0.05, see Figure 7A–C).

## 4. Discussion

In this research, HFD combined with STZ (100 mg/kg) intraperitoneal injection was used to establish a C57BL/6 mouse model of type 2 diabetic liver injury, and HepG2 cells were cultured in a 50 mM high-glucose environment for 48 h to establish a cell model, aiming to explore the protective effect and molecular mechanism of punicalagin against diabetic liver injury, so as to provide experimental fundamentals for the prevention and treatment of diabetic liver injury. Currently, most of the drugs used for the treatment of diabetic liver injury are single hypoglycemic and hepatoprotective drugs with poor efficacy and many side effects [20,21,22]. Consequently, the search for safe and effective functional active ingredients from botanicals has become a research hotspot. As the main component of pomegranate polyphenols, punicalagin has biological activities such as antioxidant, antitumor, anti-inflammatory [23,24,25], and autophagy regulation [26,27].

Referring to other studies and our previous research results [28,29], we chose 20 mg/kg of punicalagin for the intervention in in vivo experiments, and 5, 10, and 20 μM of punicalagin as low, medium, and high doses, respectively, in in vitro experiments. First, this study verified the protective effect of punicalagin against diabetic liver injury. The results showed that after 8 weeks of HFD combined with an intraperitoneal injection of STZ (100 mg/kg), the mice all showed typical symptoms of type 2 diabetes such as “three more and one less” and FBG ≥ 11.1 mmol/L, which was consistent with other studies, suggesting that the type 2 diabetes mice model was successfully established. After punicalagin intervention for 8 weeks, the typical symptoms of "three more and one less" were improved, fasting blood glucose decreased, insulin resistance level decreased, and insulin sensitivity was enhanced in the T2DM + PU group mice compared with the T2DM group mice, indicating that punicalagin has a protective effect in type 2 diabetic mice. Meanwhile, punicalagin decreased the levels of ALT and AST in the serum of type 2 diabetic mice and improved the structural disorder, fatty lesions, and inflammatory infiltration in the liver tissue of type 2 diabetic mice. In conclusion, type 2 diabetes was often accompanied by impaired liver function, and the intervention of punicalagin improved liver function in type 2 diabetic mice to some extent. The results of liver morphology and liver index further confirmed the above inferences.

Recent studies have shown that oxidative stress plays a key role in the occurrence and development of diabetic liver injury. Since punicalagin has a strong antioxidant ability, this study further explored its molecular mechanism of protecting against diabetic liver injury from the perspective of antioxidative stress. The activity of SOD can indirectly reflect the body’s ability to scavenge oxygen free radicals, while the level of MDA can indirectly reflect the severity of the attack of free radicals on the body’s cells. In this research, MDA and T-SOD levels in serum and liver tissues of mice were measured simultaneously to comprehensively observe oxidative stress levels. The results showed that punicalagin improved the activity of T-SOD in the mouse liver and reduced the MDA levels in the mouse serum and liver, indicating that punicalagin could protect against diabetic liver injury by lowering the level of oxidative stress in the liver.

This study further explored the molecular mechanism by which punicalagin alleviates the level of oxidative stress in the liver. Manganese superoxide dismutase (MnSOD) is an important antioxidant enzyme found mainly in the mitochondrial matrix of cells and is abundant in the liver, heart, kidney, and other tissues containing large amounts of mitochondria. MnSOD protects cells from oxidative stress-induced damage by scavenging excess ROS [30]. Catalase (CAT), found mainly in red blood cells and certain tissue peroxides, exerts antioxidant effects by facilitating the breakdown of hydrogen peroxide into water and oxygen [31]. In the present study, the activities of MnSOD and CAT in the serum and liver of mice in the T2DM group were noticeably reduced, and the protein expression levels of MnSOD and CAT in mice in the T2DM group and HepG2 cells in the HG group were also decreased, while the intervention of punicalagin reversed this change. These results suggest that punicalagin exerts an antioxidative stress biological function by upregulating the levels of MnSOD and CAT.

In addition, mitochondria play an influential role in cell survival and death as the main site of intracellular oxidative phosphorylation [32]. As one of the more sensitive organelles in hepatocytes, mitochondria have been found to be abnormal in structure and function in liver diseases such as type 1 and type 2 diabetic liver injury [33,34,35,36]. Mitochondrial damage is closely related to the excessive production of ROS and is not only the main site of ROS production but also an important target site for ROS action. Damaged mitochondria can further stimulate ROS production, forming a vicious circle. ROS and mitochondrial oxidative damage are the common mechanisms of many apoptosis-inducing factors. Therefore, timely cleanup of these impaired mitochondria and maintenance of mitochondrial mass and quantity balance are essential for normal cellular life activities. 

In reaction to mitochondrial injury, mitochondria can repair themselves with lysosomes, which is known as mitophagy. Some studies have confirmed the beneficial role of mitophagy in the regulation of acute and chronic liver injury. For instance, quercetin reduced hepatic steatosis by inducing mitophagy through the Pink1/Parkin signaling pathway, thereby protecting NAFLD [37]. Moreover, Zhou et al. found that melatonin could induce mitophagy through Binp3 mediated by the NR4A1/DNA-PKcs/p53 signaling pathway, thereby improving mitochondrial and liver functions in the non-alcoholic fatty liver [38]. Sun et al. showed that cadmium increased oxidative stress levels through the PINK1-Parkin pathway, damaged mitochondrial structure and function, disrupted the homeostasis of mitochondrial fission and fusion, and induced mitophagy that played a protective role in early cadmium-induced liver injury [39]. Meanwhile, it was found that NF-κB-dependent accumulation of P62/SQSTM1 induced excessive mitophagy and disrupted glucose and lipid metabolism in adipocytes [40]. Another study found that Atg7 loss in early adipose tissue led to autophagy inactivation, which increased mitochondrial content and adipogenesis and increased insulin sensitivity [41], while blocking autophagy in ripe fat tissue led to the accumulation of impairment and provoked peripheral insulin resistance [42]. Mitophagy may play different roles in different tissues at different periods. In this study, punicalagin upregulated MMP in mouse liver and HepG2 cells, and upregulated the protein expression levels of Bnip3, Pink1, Parkin, LC3b, and P62, suggesting that punicalagin may also perform a biological function of antioxidant stress by upregulating mitophagy. In addition, previous studies have shown that punicalagin can upregulate autophagy through the Akt/FoxO3a signaling pathway to alleviate diabetic liver injury, but it is unclear which organelle autophagy plays a role. This study suggests that mitophagy may play a key role in the improvement of diabetic liver injury by punicalagin, and it is necessary to further explore the relationship between mitophagy and the Akt/FoxO3a signaling pathway. 

Our results showed that punicalagin increased the expression of MnSOD and CAT in mouse liver and HepG2 cells, and upregulated MMP and the expression levels of mitophagy-related proteins such as Bnip3, Pink1, Parkin, LC3b, and P62, indicating that punicalagin may improve liver oxidative stress by enhancing the activity of the antioxidant enzyme system and upregulating mitophagy, thereby protecting diabetic liver injury. However, the interactions of the antioxidant enzyme system, mitophagy, and mitochondrial quality and quantity in the improvement of diabetic liver injury by punicalagin need to be further investigated.

## 5. Conclusions

In summary, this study verified the protective effect of punicalagin on diabetic liver injury, further explored the potential mechanism, and for the first time observed the role of mitophagy in the protective effect of punicalagin on diabetic liver injury. We provide evidence that punicalagin can reduce the level of liver oxidative stress by upregulating mitophagy and the activities of antioxidant enzymes such as MnSOD and CAT, thus playing a certain protective role in diabetic liver injury. 

## Figures and Tables

**Table 1:** Effect of punicalagin (PU) on drinking water of T2DM mice (*n* = 10).

Weeks	Control	T2DM	T2DM + PU
1	4.52 ± 0.88	12.94 ± 1.74 **	11.94 ± 1.53 **
2	4.38 ± 0.89	12.64 ± 0.76 **	11.25 ± 1.25 **^, ##^
3	4.76 ± 0.95	13.94 ± 0.86 **	11.78 ± 1.27 **^, ##^
4	4.97 ± 0.83	13.99 ± 1.41 **	11.45 ± 1.27 **^, ##^
5	4.51 ± 0.70	13.89 ± 1.71 **	11.06 ± 1.44 **^, ##^
6	5.07 ± 1.00	14.48 ± 0.79 **	10.5 ± 0.92 **^, ##^
7	4.86 ± 0.74	14.84 ± 1.18 **	9.41 ± 1.27 **^, ##^
8	4.80 ± 0.42	14.27 ± 1.40 **	8.64 ± 1.01 **^, ##^

** *p* < 0.01 vs. control (Con) group; ^##^ *p* < 0.01 vs. T2DM group.

**Table 2:** Biochemical indicator level of blood lipid level (*n* = 10).

	Control	T2DM	T2DM + Punicalagin (PU)
Serum			
TC (mmol/L)	7.18 ± 1.07	12.25 ± 1.82 **	8.42 ± 1.44 ^##^
TG (mmol/L)	0.79 ± 0.11	1.31 ± 0.25 **	1.05 ± 0.15 *^, #^
LDL-C (mmol/L)	0.22 ± 0.02	0.67 ± 0.21 **	0.26 ± 0.13 ^##^
FFA (μmol/L)	849.4 ± 50.0	964.7 ± 89.7 *	845.7 ± 39.6 ^#^
Liver tissue			
TC (mmol/gprot)	0.09 ± 0.01	0.21 ± 0.03 **	0.13 ± 0.04 *^, ##^
TG (mmol/gprot)	0.2 ± 0.02	1.33 ± 0.63 **	0.61 ± 0.32 ^#^
LDL-C (mmol/gprot)	0.02 ± 0.01	0.09 ± 0.04 *	0.02 ± 0.01 ^#^
FFA (μmol/L)	1140.6 ± 165.5	1351.8 ± 91.6 *	1147.2 ± 89.3 ^#^

* *p* < 0.05 vs. Con group; ** *p* < 0.01 vs. Con group; ^#^ *p* < 0.05 vs. T2DM group; ^##^ *p* < 0.01 vs. T2DM group.
